# Complete genome sequence of a novel species of *Brevundimonas* (strain NIBR10)

**DOI:** 10.1128/MRA.00265-23

**Published:** 2023-07-21

**Authors:** Myeong-Hyeon Min, Dong-Hyun Jung, Jin Nam Kim, Jaewoong Yu, Ahhyeon Choi

**Affiliations:** 1 Department of Plant Resources, College of Industrial Science, Kongju National University, Yesan-gun, Republic of Korea; 2 Microorganism Resources Division, National Institute of Biological Resources, Incheon, Republic of Korea; 3 eGnome, Inc, Seoul, Republic of Korea; Wellesley College, Wellesley, Massachusetts, USA

**Keywords:** genome assembly, prokaryote

## Abstract

The complete genome sequence of strain NIBR10 was sequenced using PacBio RS II (Pacific Biosciences) sequencing platform. The 4,006,378-bp genome has a G + C content of 66.89% and around 3,832 coding sequences. Genomic data will provide valuable research for natural taxonomy and comparative genomics of the genus *Brevundimonas*.

## ANNOUNCEMENT

The genus *Brevundimonas* was re-classified by Segers et al. (1). *Brevundimonas* belong to Gram-negative and non-fermenting bacteria. *Brevundimonas* can survive in various conditions (2). The significant conditions associated with *Brevundimonas* spp. are increasing, although *Brevundimonas* spp. are considered rare clinical instances (3). Strain NIBR10 was isolated from a mud sample from swamp land in the Namdong area. Namdong area is a mountainous area with Hambaksan Mountain and Nogobong Mountain located in Yongin-si in the Republic of Korea (37°13′14.0″N 127°13′10.0″E).

Biomass was grown in R2A medium by the standard dilution-plating method. These plates were incubated at 25°C for 3 d, and colonies were streaked to obtain a pure culture. A single colony was cultured under the same condition and was preserved at −80°C in 20% glycerol stock. Also, the single colony was obtained again from the frozen stock through streaking. The 16S rRNA gene amplification was carried out following standard procedures by Woodman et al. (4) with universal primer sets (27F/1492R) (5). The 16S rRNA PCR product was sequenced by the Sanger method on Applied Biosystems automatic Sequencer 3730XL (Thermo Fisher Scientific, Applied Biosystems, Waltham, MA, USA). The 16S rRNA gene sequence was searched by BLASTn against the NCBI 16S rRNA database and confirmed as *Brevundimonas staleyi* FWC43 (NR_114710.1) with 99.0% identity. Genomic DNA was extracted by an RBC DNA extraction kit (RBC Bioscience, Taiwan) and sheared to over 15 kb by the Covaris g-TUBE (Covaris, Woburn, MA, USA). The sheared DNA was cleaned using AMPure XP beads (Beckman Coulter, Danvers, MA, USA) and measured by Bioanalyzer (Agilent, Santa Clara, CA, USA). The library was prepared using PacBio SMRTbell library preparation kit version 1.0 (Pacific Biosciences, Menlo Park, CA, USA) and size selected using the BluePippin (Sage Science, Beverly, MA, USA) with a 15-kb cutoff. The size-selected library was sequenced on the PacBio RS II platform using Pacbio P6-C4 chemistry.

**Fig 1:**
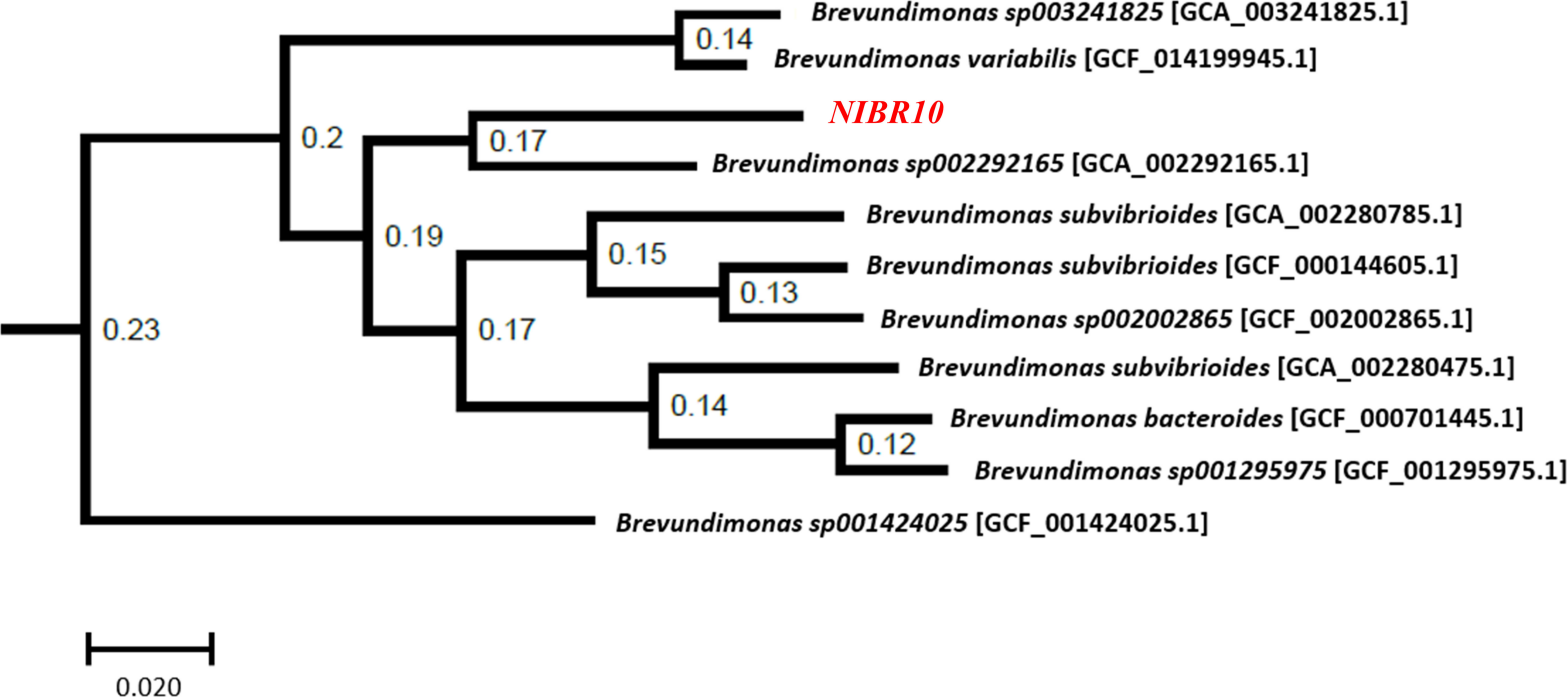
Phylogenetic tree based on the strain NIBR10 and GTDB-tk r202 *Brevundimonas* species representatives. The GTDB-tk de_novo_wf workflow generated a multiple-sequence alignment.

After sequencing, 2,601,563 reads were produced with a total length of 17,186,990,103 bp and an N50 value of 8,172 bp. Pacbio raw reads less than 1,000 bp were filtered using SeqKit version 2.3.0 (6). The filtered reads were assembled using Flye version 2.8.1 (7) with the “-meta” option. Default parameters were used for all software unless otherwise specified. The assembled genome was rotated to start at dnaA using the fixstart method in Circlator version 1.5.5 (8). Finally, one circular chromosome of 4,006,378 bp is obtained. The quality of assembled genome was assessed with QUAST version 5.0.2 (9) and BUSCO version 5.2.2 (10). The strain NIBR10 genome had a BUSCO completeness score of 99.2% with the bacteria_odb10 data set. Genome annotation was performed using Prokka version 1.14.6 (11). Features in the NIBR10 genome include a G + C content of 66.89%, and around 3,832 coding sequences, 6 of which are suggested to encode rRNAs and 49 of which are suggested to encode tRNAs. GTDB-Tk version 1.5.0 was used for a taxonomic assignment using the release 202 (12). GTDB-Tk identified strain NIBR10 as a new species in the genus *Brevundimonas*. Also, strain NIBR10 shared the highest ANIb value (82.85%) and was most closely related to *Brevundimonas sp002292165* (GCA_002292165.1) (Fig. 1).

## Data Availability

The draft genome sequence of strain NIBR10 has been deposited in GenBank under the accession number CP115464. The raw data have been deposited in the SRA under the accession number SRR22876612.

## References

[1] Segers P , Vancanneyt M , Pot B , Torck U , Hoste B , Dewettinck D , Falsen E , Kersters K , De Vos P . 1994. Classification of Pseudomonas diminuta Leifson and Hugh 1954 and Pseudomonas vesicularis Büsing, Döll, and Freytag 1953 in Brevundimonas gen. nov. as Brevundimonas diminuta comb. nov. and Brevundimonas vesicularis comb. nov., respectively. Int J Syst Bacteriol 44:499–510. doi:10.1099/00207713-44-3-499 8068543

[2] Liu L , Feng Y , Wei L , Zong Z . 2021. Genome-Based Taxonomy of Brevundimonas with reporting Brevundimonas huaxiensis sp. nov. Microbiol Spectr 9:21–e00111. doi:10.1128/Spectrum.00111-21 PMC8552745 34232096

[3] Ryan MP , Pembroke JT . 2018. Brevundimonas spp: emerging global opportunistic pathogens. Virulence 9:480–493. doi:10.1080/21505594.2017.1419116 29484917 PMC5955483

[4] Woodman ME . 2008. Direct PCR of intact bacteria (colony PCR). Curr Protoc Microbiol 9:A. 3D. 1-A. 3D. 6. doi:10.1002/9780471729259.mca03ds9 27517337

[5] Lane D . 1991. 16S/23S rRNA sequencing. nucleic acid techniques in bacterial systematics

[6] Shen W , Le S , Li Y , Hu F . 2016. Seqkit: a cross-platform and ultrafast toolkit for FASTA/Q file manipulation. PLoS One 11:e0163962. doi:10.1371/journal.pone.0163962 27706213 PMC5051824

[7] Kolmogorov M , Yuan J , Lin Y , Pevzner PA . 2019. Assembly of long, error-prone reads using repeat graphs. Nat Biotechnol 37:540–546. doi:10.1038/s41587-019-0072-8 30936562

[8] Hunt M , Silva ND , Otto TD , Parkhill J , Keane JA , Harris SR . 2015. Circlator: automated circularization of genome assemblies using long sequencing reads. Genome Biol 16:294. doi:10.1186/s13059-015-0849-0 26714481 PMC4699355

[9] Gurevich A , Saveliev V , Vyahhi N , Tesler G . 2013. QUAST: quality assessment tool for genome assemblies. Bioinformatics 29:1072–1075. doi:10.1093/bioinformatics/btt086 23422339 PMC3624806

[10] Manni M , Berkeley MR , Seppey M , Simão FA , Zdobnov EM , Kelley J . 2021. BUSCO update: novel and streamlined workflows along with broader and deeper phylogenetic coverage for scoring of eukaryotic, prokaryotic, and viral genomes. Mol Biol Evol 38:4647–4654. doi:10.1093/molbev/msab199 34320186 PMC8476166

[11] Seemann T . 2014. Prokka: rapid prokaryotic genome annotation. Bioinformatics 30:2068–2069. doi:10.1093/bioinformatics/btu153 24642063

[12] Chaumeil P-A , Mussig AJ , Hugenholtz P , Parks DH . 2020. GTDB-TK: A Toolkit to classify Genomes with the genome Taxonomy database. Oxford University Press. doi:10.1093/bioinformatics/btz848 PMC7703759 31730192

